# Supplementary material for: Association of autoantibodies with the IFN signature and NETosis in patients with systemic lupus erythematosus
Source: J Transl Autoimmun. 2024 Jun 20;9:100246. doi: 10.1016/j.jtauto.2024.100246 (PMC11254743; doi:10.1016/j.jtauto.2024.100246)
Supplement: Multimedia component 1 [file mmc1.docx]

**Association of autoantibodies with the IFN signature and NETosis in patients with Systemic Lupus Erythematosus**

Ellen D. Kaan^a,b,c,1^, Tammo E. Brunekreef^b,1^, Julia Drylewicz^a^, Lucas L. van den Hoogen^a,b,2^, Maarten van der Linden^a,3^, Helen L. Leavis^a,b^, Jacob M. van Laar^b^, Michiel van der Vlist^a,c^, Henny G. Otten^a,d^, Maarten Limper^b*^

*^a^Center for Translational Immunology, University Medical Center Utrecht, Utrecht University, Utrecht, The Netherlands*

*^b^Department of Rheumatology & Clinical Immunology, University Medical Center Utrecht, Utrecht University, Utrecht, The Netherlands*

*^c^Oncode Institute, Utrecht, The Netherlands*

*^d^Central Diagnostic Laboratory, University Medical Center Utrecht, Utrecht University, Utrecht, The Netherlands*

*Corresponding author: Maarten Limper, Department of Rheumatology & Clinical Immunology, University Medical Center Utrecht, P.O. Box 85500, 3508 GA, Utrecht, The Netherlands, m.limper-2@umcutrecht.nl
^1^These authors contributed equally to this article
^2^Present affiliation: department of Rheumatology, Radboudumc and Sint Maartenskliniek, Nijmegen, The Netherlands
^3^Present affiliation: Citryll B.V., Oss, The Netherlands.

**Highlights**

- We describe the association between autoantibodies, the IFN signature, NETosis, and clinical features in patients with SLE.
- A subgroup of patients expresses higher concentrations of DNA- and RNA-binding autoantibodies. This is associated with a high IFN signature, and cutaneous rash.
- An autoantibody profile might be used for patient stratification, risk prediction and targeted treatment in SLE.

**Abstract
Objective** Systemic lupus erythematosus (SLE) is an autoimmune disease characterized by a variety of disease symptoms and an unpredictable clinical course. To improve treatment outcome, stratification based on immunological manifestations commonly seen in patients with SLE such as autoantibodies, type I interferon (IFN) signature and neutrophil extracellular trap (NET) release may help. It is assumed that there is an association between these immunological phenomena, since NET release induces IFN production and IFN induces autoantibody formation via B-cell activation. Here we studied the association between autoantibodies, the IFN signature, NET release, and clinical manifestations in patients with SLE.

**Methods** We performed principal component analysis (PCA) and hierarchical clustering of 57 SLE-related autoantibodies in 25 patients with SLE. We correlated each autoantibody to the IFN signature and NET inducing capacity.

**Results** We observed two distinct clusters: one cluster contained mostly patients with a high IFN signature. Patients in this cluster often present with cutaneous lupus, and have higher anti-dsDNA concentrations. Another cluster contained a mix of patients with a high and low IFN signature. Patients with high and low NET inducing capacity were equally distributed between the clusters. Variance between the clusters is mainly driven by antibodies against histones, RibP2, RibP0, EphB2, RibP1, PCNA, dsDNA, and nucleosome. In addition, we found a trend towards increased concentrations of autoantibodies against EphB2, RibP1, and RNP70 in patients with an IFN signature. We found a negative correlation of NET inducing capacity with anti-FcER (r= -0.530; p=0.007) and anti-PmScl100 (r= -0.445; p=0.03).

**Conclusion** We identified a subgroup of patients with an IFN signature that express increased concentrations of antibodies against DNA and RNA-binding proteins, which can be useful for further patient stratification and a more targeted therapy. We did not find positive associations between autoantibodies and NET inducing capacity. Our study further strengthens the evidence of a correlation between RNA-binding autoantibodies and the IFN signature.

**Keywords**Systemic lupus erythematosus; autoantibodies; interferon signature; NETosis; patient stratification;

**Abbreviations**

ANA Antinuclear antibody
AZA Azathioprine
BICLA BILAG-based Composite Lupus Assessment
BILAG British Isles Lupus Assessment Group
dsDNA Double stranded DNA
ENA Extractable nuclear antibody
EphB2 Ephrin type-B receptor 2
FcER Fc epsilon receptor
HCQ Hydroxychloroquine
IFN Interferon
MMF Mycophenolate mofetil
NET Neutrophil extracellular trap
PCA Principal component analysis
PCNA Proliferating cell nuclear antigen
PmScl100 Polymyositis/Scleroderma 100
RibP Ribosomal protein P
RNP Ribonucleoprotein
SELENA-SLEDAI Safety of Estrogens in Lupus Erythematosus National Assessment - SLEDAI
SLE Systemic lupus erythematosus
SLEDAI Systemic lupus erythematosus disease activity index
Sm Smith
SSA Sjogren’s syndrome antigen A

SSB Sjogren’s syndrome antigen B
TLR Toll-like receptor

**1. Introduction**

Systemic lupus erythematosus (SLE) is an autoimmune disease characterized by a variety of disease symptoms and an unpredictable clinical course. While some patients experience a relatively mild disease, others develop a complicated disease course, with frequent flares that can precede organ damage. Ultimately, SLE can lead to premature death, as the result of disease activity or because of treatment side effects [1]. This underlines the urgency to identify patients that develop a complicated disease course, and the need for tailored therapy [2, 3].

Risk stratification is a tool to predict progression to severe disease. In SLE a clinically applicable stratification tool or biomarker still remains high on the agenda [2]. Several cytokine phenotypes and gene signatures have been proposed as a tool for risk stratification, but these have not found their way to clinical practice yet [4-9]. The main immunological mechanisms in SLE are formation of autoantibodies, the presence of an interferon (IFN) signature, and development of neutrophil extracellular traps (NETs) [10]. Autoantibodies are a nearly omnipresent immunological manifestation in SLE patients. Antinuclear antibodies are present in most patients, although there is individual variation in the specific autoantigens that these autoantibodies recognize. Some autoantibodies are associated with specific disease manifestations. For example, anti-C1q antibodies are associated with lupus nephritis, and a rise in the serum concentration of anti-dsDNA antibodies is often seen in patients during flares [11, 12].

Besides autoantibody production, approximately half of all SLE patients show increased expression of genes regulated by proinflammatory type 1 IFNs, known as the IFN signature [13, 14]. The IFN signature is often used as a proxy for the presence of type 1 IFN, considering the absence of a reliable method to measure type 1 IFNs in vivo. Type 1 IFNs play an important role in the pathophysiology of SLE, as randomized controlled trials show that treatment with type 1 IFN receptor blocking antibody anifrolumab leads to a decrease on the British Isles Lupus Assessment Group (BILAG)-based Composite Lupus Assessment (BICLA) in patients with SLE [15, 16]. In these trials, anifrolumab suppresses interferon regulated genes in patients with a high IFN signature. Surprisingly, treatment effect was observed in patients with a high IFN signature as well as in patients with a low IFN signature. This indicates that SLE activity is not solely based on the presence of type I IFNs, and that the contribution of an IFN signature to disease activity differs between patients. Unfortunately, the small sample size of IFN low patients did not allow a direct comparison of the therapeutical effect of anifrolumab between patients with a low and high IFN signature. More information is therefore needed to determine the use of the IFN signature for personalized treatment.

NETs are important drivers of type I IFN production and a source of auto-antigens in patients with SLE [17]. NETs are formed as a first line defense mechanism of neutrophilic granulocytes by physically capturing microbes in structures containing DNA and antimicrobial peptides via a process called NETosis. NETs are relatively rich in antigens for autoantibodies commonly seen in SLE, such as double-stranded DNA. Indeed, NETosis is associated with anti-nuclear and anti-dsDNA antibodies, and the IFN signature in SLE [18]. The extent to which these immunological mechanisms and pathways are involved in the pathophysiology of SLE differs between patients.

Since NETs are a rich source of antigens for autoantibodies, and NETosis induces IFNα production, it is expected that these immunological mechanisms in SLE would to some extent correlate with each other. Indeed, NETosis is associated with the IFN signature, ANA and anti-dsDNA formation in SLE and type 1 IFNs are known to affect B-cell function and survival, which could lead to increased autoantibody production [18-20]. In the present study, we explored correlations between a wide range of established and lesser known SLE-associated autoantibodies and the presence of an IFN signature and NET release to further study their complex interaction in patients with SLE. Better understanding of these underlying immunological manifestations and their correlations could lead to better tools for patient stratification and more targeted treatment options.

**2. Materials and methods
*2.1 Patients***

Blood samples and clinical data of patients with SLE as well as healthy controls were collected in the University Medical Center (UMC) Utrecht, The Netherlands in 2014 and 2015. The study protocol was approved by the medical ethical committee of the UMC Utrecht (reference number 12-296). All participating patients provided written informed consent. All patients fulfilled the American College of Rheumatology 1997 criteria for SLE. None of the included patients had signs of infection at the time of sample collection. Patients did not receive B lymphocyte suppressive therapy (belimumab or rituximab) in the 6 months prior to inclusion in this study. The IFN signature and NET inducing capacity were determined in patients from this cohort and were previously published [18, 21]. Serum samples from these patients were stored in the UMC Utrecht biobank.

In 2019, we tested the presence of 57 SLE-related autoantibodies in biobanked serum of 483 patients with SLE, using a custom-made antibody microarray system [22]. 25 of these patients were also included in the previously reported cohort. We combined the datasets for the present study. Figure 1 shows a visual representation of the inclusion process.

***2.2 Assessment of IFN signatures, NET inducing capacity and autoantibodies***

A complete description of the methods for determining the IFN signature and NET inducing capacity has been previously published [18, 21]. In short, mRNA expression of four IFN regulated genes (IFI44L, IFITM1, SERPING1 and LY6E) was determined relative to housekeeping gene GUSB in CD14+ monocytes from SLE patients and healthy controls. The relative gene expression was normalized against expression in a group of healthy controls (HC), and the individual z-scores were averaged to determine the IFN signature. The IFN signature was considered high if the averaged z-score was above 2SD of the mean IFN signature in the HD group. NET inducing capacity was studied by stimulating healthy donor neutrophils with 10% plasma from SLE patients for 4 hours. NET inducing capacity was measured with quantitative live imaging. Youden’s J-statistic of the receiver operating characteristics (ROC) curve of NET-release in patients with SLE compared to HC’s was used to determine whether a patient has a high or low NET inducing capacity.

We determined serum concentrations of 57 IgG autoantibodies with a custom-made antigen microarray system, using indirect immunofluorescence (Thermo Fisher Scientific, Uppsala Sweden). A full description of the methods has been described previously [22].

***2.3 Statistical analysis***

We performed a principal component analysis to reduce the dimensionality of SLE patients with a high or low IFN signature, and a high or low amount of NET release, followed by a k-mean clustering approach (using principal components explaining 90% of the variance in the data) to define clusters.

We also performed a hierarchical clustering analysis to confirm the clusters, using the furthest neighbor clustering method, measuring the squared Euclidean distance of the autoantibodies measured on the antibody microarray. The scores of immunofluorescent intensity were log transformed and expressed as Z-scores to correct for differences in mean concentrations between the different autoantibodies. Data were visualized in a heatmap. Hierarchical clustering and principal component analysis were performed in R (www.r-project.org, version 4.2.2).

We used Mann-Whitney-U tests to compare concentrations of immunofluorescence of different autoantibodies between patients with high and low scores for IFN signature and NET inducing capacity. P-values were corrected for multiple testing according to the Bonferroni Correction (α = 0.05/57 = 0.0009). We used Spearman’s correlation to analyze the relationship between IFN signature, NET inducing capacity and concentrations of autoantibodies. Statistical analysis was performed using SPSS (version 25.0.0.2). Figures were made using GraphPad Prism (version 8.3.0).

**3. Results**

***3.1 Baseline characteristics***

The median age of all included patients was 43 years, 92% of the patients were female. Most patients were in remission or had low disease activity at the time of sample collection, although three patients had active disease (SLEDAI 6, 9 and 16). Nearly all patients (96%) tested positive for anti-nuclear antibodies (ANA). At the time of inclusion, six patients (24%) tested positive for anti-double stranded DNA antibodies (anti-dsDNA), although 22 patients (88%) had ever tested positive for anti-dsDNA. Nine patients (36%) had decreased concentrations of C3 and/or C4. A full overview of the baseline characteristics is presented in table 1.

Patients with a high IFN signature, or high NET inducing capacity had a higher median SLEDAI and anti-dsDNA concentrations, whereas median complement concentrations in these groups were lower compared to their counterparts with low IFN signature and low NET inducing capacity. All three patients who never tested positive for anti-dsDNA had a low IFN signature and low NET inducing capacity.
Patients with a high IFN signature and/or high NET inducing capacity were more likely to have active disease as well as receive medication other than hydroxychloroquine (HCQ).

**Table 1:** Patient characteristics

|  |  | **All patients** | **IFN signature** | | | **NET inducing capacity** | |
| --- | --- | --- | --- | --- | --- | --- | --- |
|  |  | **N=25** | **Low**  **n=7** | **High**  **n=15** | **Missing**  **n=3** | **Low**  **n=10** | **High**  **n=15** |
| **Age in years**^a^ |  | 43 (21-65) | 43 (21-59) | 44 (22-65) |  | 43 (21-59) | 43 (25-65) |
| **Female sex (%)** |  | 23 (92%) | 7 (100%) | 14 (93%) |  | 10 (100%) | 13 (87%) |
| **SELENA-SLEDAI score**  **at inclusion**^a^ |  | 2 (0-16) | 0 (0-2) | 2 (0-16) |  | 1 (0-4) | 2 (0-16) |
| **Serology** |  |  |  |  |  |  |  |
| *ANA positive (%)* |  | 24 (96%) | 6 (86%) | 15 (100%) |  | 9 (90%) | 15 (100%) |
| *ENA positive (%)* |  | 17 (68%) | 3 (43%) | 13 (93%) |  | 6 (60%) | 11 (73%) |
| *Anti-dsDNA (IU/mL)*^a^ |  | 6.1 (0.9-497.0) | 1.6(0.9-21.0) | 8.7 (1.5-497.0) |  | 1.9 (0.9-27.0) | 8.2 (1.5-497.0) |
| *Anti-dsDNA ever positive* |  | 22 (88%) | 4 (57%) | 15 (100%) |  | 7 (70%) | 15 (100%) |
| *C3(g/L)* ^a^ |  | 0.99 (0.54-1.51) | 1.09 (0.96-1.51) | 0.89 (0.64-1.41) |  | 1.08 (0.68-1.51) | 0.92 (0.54-1.41) |
| *C4 (g/L)* ^a^ |  | 0.18 (0.06-0.34) | 0.22 (0.18-0.34) | 0.16 (0.06-0.25) |  | 0.19 (0.11-0.34) | 0.18 (0.06-0.25) |
| **Current use of**  **immunosuppressive**  **medication (%)** | *HCQ* | 18 (72%) | 5 (71%) | 11 (73%) |  | 7 (70%) | 11 (73%) |
|  | *Prednisone* | 9 (36%) | 1 (14% | 6 (40%) |  | 3 (30%) | 6 (40%) |
|  | *AZA* | 7 (28%) | 1 (14%) | 4 (27%) |  | 3 (30%) | 4 (27%) |
|  | *MMF* | 1 (4%) | 0 | 1 (7%) |  | 0 | 1 (7%) |

^a^Categorical variables are represented as medians with range. SELENA-SLEDAI: Safety of Estrogens in Lupus Erythematosus National Assessment – Systemic Lupus Erythematosus Disease Activity Index; ANA: antinuclear antibodies; ENA extractable nuclear antigen antibodies; anti-dsDNA: anti-double-stranded DNA; HCQ: hydroxychloroquine; AZA: azathioprine; MMF: mycophenolate mofetil; IFN: interferon; NET: neutrophil extracellular traps.

***3.2 A distinct autoantibody pattern is present in a subgroup of patients with a high IFN signature, and is associated with development of a new rash***
To investigate if patients with a IFN signature and/or NETosis have a specific autoantibody profile, we performed a principal component analysis (PCA) followed by a k-mean clustering (Fig. 2A). We found an undetectably low concentration of citrullinated α-enolase antibodies in one patient. This unusual value was defined as an outlier and consequently excluded from further clustering analyses. We tested whether excluding this patient influenced the clustering analyses, and found that the results represented in figure 1 are not dependent of this patient. An overview of the studied autoantibodies is included as supplementary table 1. We identified two distinct clusters: cluster 1 (n=9) contains mostly patients with a high IFN signature, and cluster 2 (n=15) contains a mix of patients with a high and low IFN signature. The two clusters are mainly separated by PC1 explaining 22.7% of the total variability. PC1 is mainly driven by antibodies against histones, RibP2, RibP0, EphB2, RibP1, PCNA, dsDNA, and nucleosome. The component loadings for PC1, PC2 and PC3 are shown in supplemental figure S1. A hierarchical cluster analysis shows two distinct clusters, and one patient previously clustering with patients in cluster 1, crossed over to cluster 2. Hierarchical clustering confirmed the clustering of patients with a distinct autoantibody pattern and a high IFN signature (Fig. 2B).
We evaluated whether patients presented with different clinical features and found an increased median anti-dsDNA and thrombocyte concentration in cluster 1 compared to cluster 2 (Fig. 2C). Although the median SELENA-SLEDAI score did not differ, cluster 1 contained all four patients that developed a new rash (Fig. 2D). Other clinical SELENA-SLEDAI features did not significantly differ between the clusters.

***3.3 Autoantibodies against RibP1, RNP70 and EphB2 are associated with the IFN signature***We further analyzed the association between specific autoantibodies and the IFN signature. We compared the autoantibody concentration of patients with a high IFN signature to the concentration of patients with a low IFN signature and found a trend towards increased concentrations of autoantibodies against EphB2, RibP1, and RNP70 in patients with a high IFN signature (Figure 3A). Moreover, we found positive correlations between EphB2, RipP1, and RNP70 and the IFN signature (Figure 3B).

***3.4 Categorizing patients based on NET inducing capacity does not reveal distinct autoantibody clusters***When we categorized patients based on NET inducing capacity, we did not identify clustering of patients with certain autoantibody profiles (Fig. 2B). We additionally compared autoantibody concentrations in patients with a high NET inducing capacity to concentrations in patients with a low NET inducing capacity. Patients with a high NET inducing capacity tend to have decreased concentrations of antibodies against FcER and PmScl100 compared to patients with a low NET inducing capacity (Figure 4A). Antibodies against FcER and PmScl100 showed a negative correlation to NETosis (Figure 4B).

**4. Discussion**In this study, we investigated the association between the IFN signature, NETosis and 57 autoantibodies in SLE patients, since further insight in the interplay of these immunological manifestations could lead to better options for patient stratification and personalized treatment.
Stratifying patients with SLE based on underlying immunological traits has gained attention in recent years, and has been attempted in several cohorts [23-27]. The use of different autoantibody panels and sample sizes however impairs comparability between studies. Recently, a clustering analysis based on 20 autoantibodies and 29 ANA patterns in a large cohort of patients with SLE identified four distinct autoantibody clusters [28]. One cluster contained patients positive for antibodies against (among others) dsDNA, RibP, histones, and PCNA. These antibodies were among the main drivers of variability in our PCA.

We found a subgroup of patients with an IFN signature who express increased concentrations of antibodies against DNA and RNA-binding proteins. In line with our findings, others have previously described an association between antibodies against RibP0, RNP, anti-dsDNA, and the IFN signature [29-31]. However, we could not confirm previously proposed associations between the IFN signature and anti-SSA, anti-SSB, and anti-Sm [31].

The exact interplay of RNA-binding antibodies and the IFN signature is not fully understood. Interestingly, RNA-binding antibodies are more prone to forming circulating immune complexes than DNA-binding antibodies in SLE patients, which are more likely to bind directly to the target tissue [32]. As immune complexes are thought to be an important source of IFN via signaling through TLR7 and TLR9, this suggests that the RNA-related pathway involving TLR7 is a driver of IFN production in patients with SLE. Whether RNA-binding autoantibodies such as anti-U1 RNP, of which RNP70 is a subcomponent, play a causative role in the pathophysiology or are an epiphenomenon remains to be elucidated.

We found a trend towards increased concentrations of autoantibodies against EphB2, RibP1, and RNP70 in IFN-positive patients. Especially the presence of antibodies against the U1 RNP complex is associated with an interferon signature [33, 34]. This association is not only seen in SLE, but across systemic autoimmune diseases, indicating that subgroups of patients with systemic autoimmunity share immunopathological phenotypes, despite different clinical disease manifestations [35, 36]. The association between antibodies against ribosomal protein RibP1 and the IFN signature is less studied, although patients with anti-RibP antibodies have higher serum concentrations of IFN alpha than patients who were negative for anti-RibP antibodies [37].

To our surprise, we found that the presence of antibodies directed against EphB2 is one of the main drivers of variability between patients with a high IFN signature and patients with a low IFN signature. We found a positive correlation between antibodies against EphB2 and the IFN signature, in addition to a previously described correlation between EphB2 protein and the IFN signature [38]. EphB2 protein is involved in atherosclerosis, and EphB2 expression in atherosclerotic plaques increases with plaque severity [39, 40]. Antibodies against EphB2 have been observed in both SLE and systemic sclerosis patients. SLE patients with anti-EphB2 antibodies more often experience cardiovascular events compared to patients that do not express this antibody [41]. Whether EphB2, or antibodies against EphB2, play a causative role in atherosclerotic plaque formation in SLE is to be further elucidated, but its presence might partially explain the observed increased prevalence of cardiovascular events in patients with SLE compared to healthy patients [42].

As NETs are rich sources of antigens for antibodies commonly seen in SLE, we expected a positive association between NETosis and several of these autoantibodies. Indeed, an association between NETosis and anti-dsDNA has been reported [43] and was also found in the original cohort from which this sub analysis was performed [18]. Here, we did not find positive associations of autoantibodies with NETosis, but rather found a negative correlation of NETosis with anti-FcER and anti-PmScl100, indicating that we performed analyses for this present study in a subgroup from the original cohort. Although anti-FcER and anti-PmScl100 are mostly associated with chronic urticaria and systemic sclerosis respectively, both have also been described in patients with SLE [44, 45].

The main limitation in our study is the small sample size: we associated 57 autoantibodies with NETosis and the IFN signature in 25 and 22 patients respectively. For these analyses, we combined data from the same patients that were part of both studies, but we did not perform a power calculation beforehand. The low number of patients with active disease and the cross-sectional design of the study limits predicting flares and does not allow us to identify patients at risk for a complicated disease course. However, our findings are in line with current literature, indicating that our cohort might not be large enough to detect statistically significant differences after correction for multiple testing, but is still representative for SLE patients.

**5. Conclusion**

In this study, we show that combining data on different immunological phenomena in patients with SLE can help to further stratify patients. We identified a subgroup of patients with a high IFN signature that express DNA and RNA-binding antibodies, as well as antibodies against EphB2. Larger observational cohort studies are needed to further stratify patients based on a combination of genetic, proteomic and autoantibody profiles. This could lead to improved prediction of flares in SLE, as well as more targeted treatment options.

**Ethics approval**This study was conducted in accordance with the Declaration of Helsinki as revised in 2013. The medical ethical committee of the UMC Utrecht approved the study protocol (reference number 12-296). Use of serum samples was approved by the biobank committee of the UMC Utrecht (Toetsingscommissie Biobanken UMC Utrecht, TCBio protocol no. 16-824).

**Funding**This work was supported by the Dutch Arthritis Society, grant number 18-1-403.

**CRediT authorship contribution statement**EK and TB performed data collection, analysis, interpretation and visualization. EK and TB wrote the manuscript. JD performed PCA and hierarchical clustering. LvdH determined the IFN signature. MvdL and LvdH measured plasma induced NET capacity. HL and JvL provided input on the data interpretation. MvdV, HO and ML conceptualized and designed the study, interpreted the data, wrote the manuscript, and provided overall supervision. All authors critically reviewed the manuscript. ML acts as guarantor for this study.

**Declaration of competing interest**JvL reports financial support was provided by Thermofischer. JvL reports a relationship with Abbvie, Eli Lilly, Galapagos, Magenta. that includes: consulting or advisory and funding grants. All other authors have no competing interests to declare.

**Data availability statement**The data that support the findings presented in this study are available from the corresponding author upon request.

**Figures**


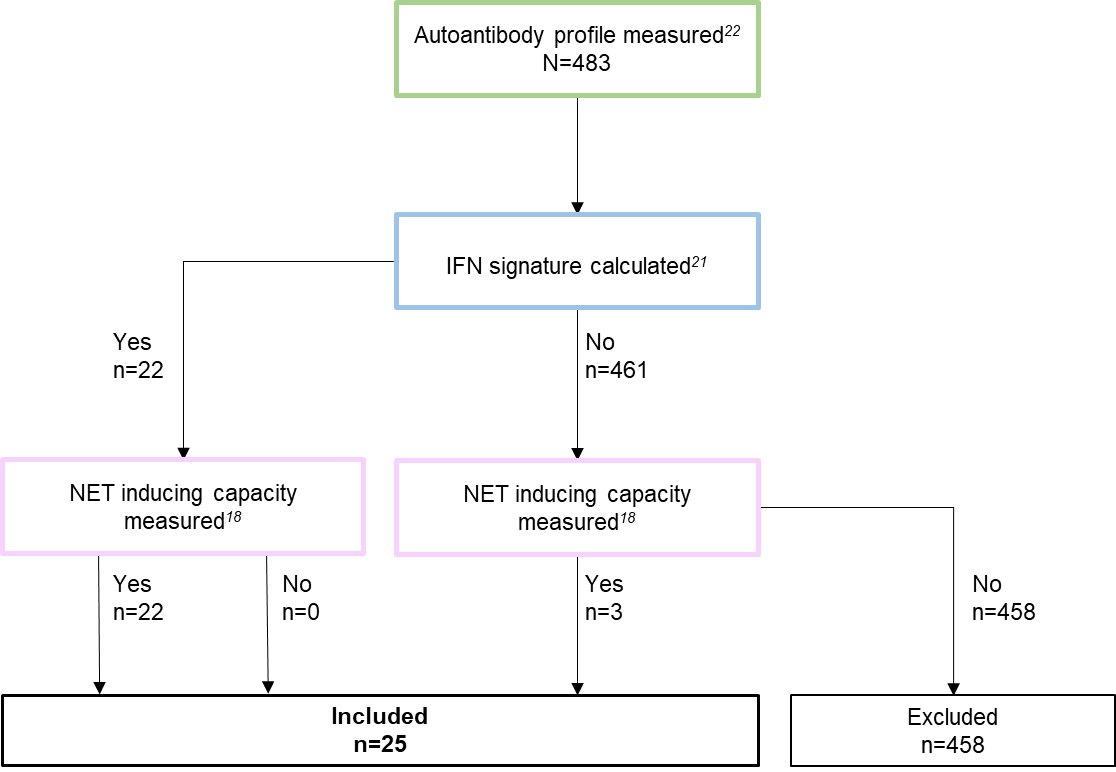


**Figure 1:** Flowchart representing the patient selection process. IFN: interferon; NET: neutrophil extracellular traps.

**
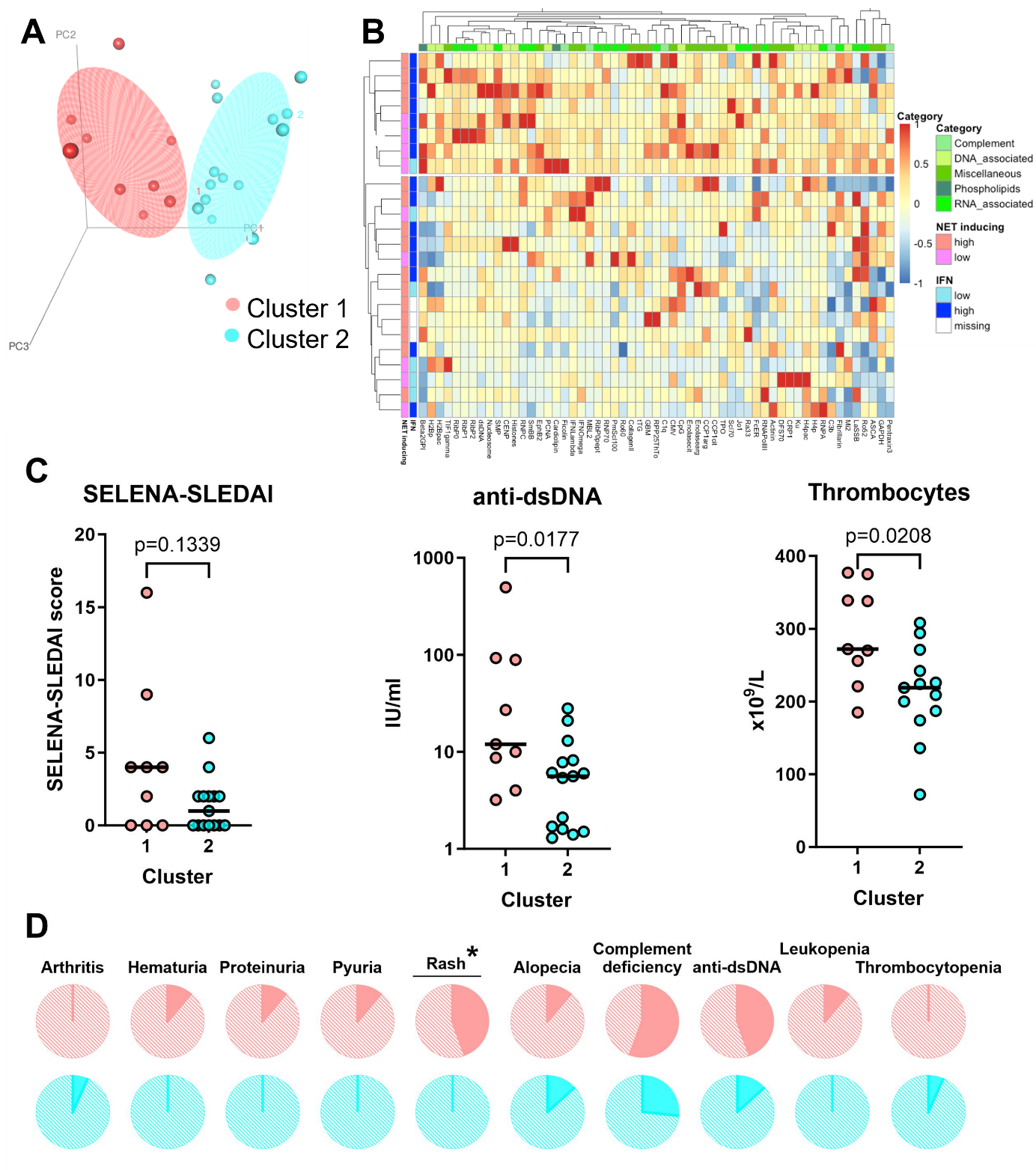
**

**Figure 2:** **(A)** Principal component analysis of 24 patients with SLE visualized in a 3D plot. **(B)** Heatmap of Z-scores for 57 autoantibodies in patients with high and low IFN signature, and high or low NET inducing capacity. Vertical axis shows clustering of patients based on IFN status (blue) and NET inducing capacity (orange/pink). Horizontal axis represents clustering based on autoantibody profiles. **(C)** SELENA-SLEDAI score, anti-dsDNA, and thrombocyte concentration in cluster 1 and 2 from figure 2A. Lines represent median. Differences between clusters were tested with a Mann-Whitney-U test. **(D)** Representation of individual SELENA-SLEDAI items in patients in cluster 1 and cluster 2 from figure 2A. Darker area represents % of patients positive for this item. Only items present in patients from our cohort are shown. *χ^2^(=1, N=24) =8.00, p=0.005. IFN: interferon signature; NET: neutrophil extracellular trap.


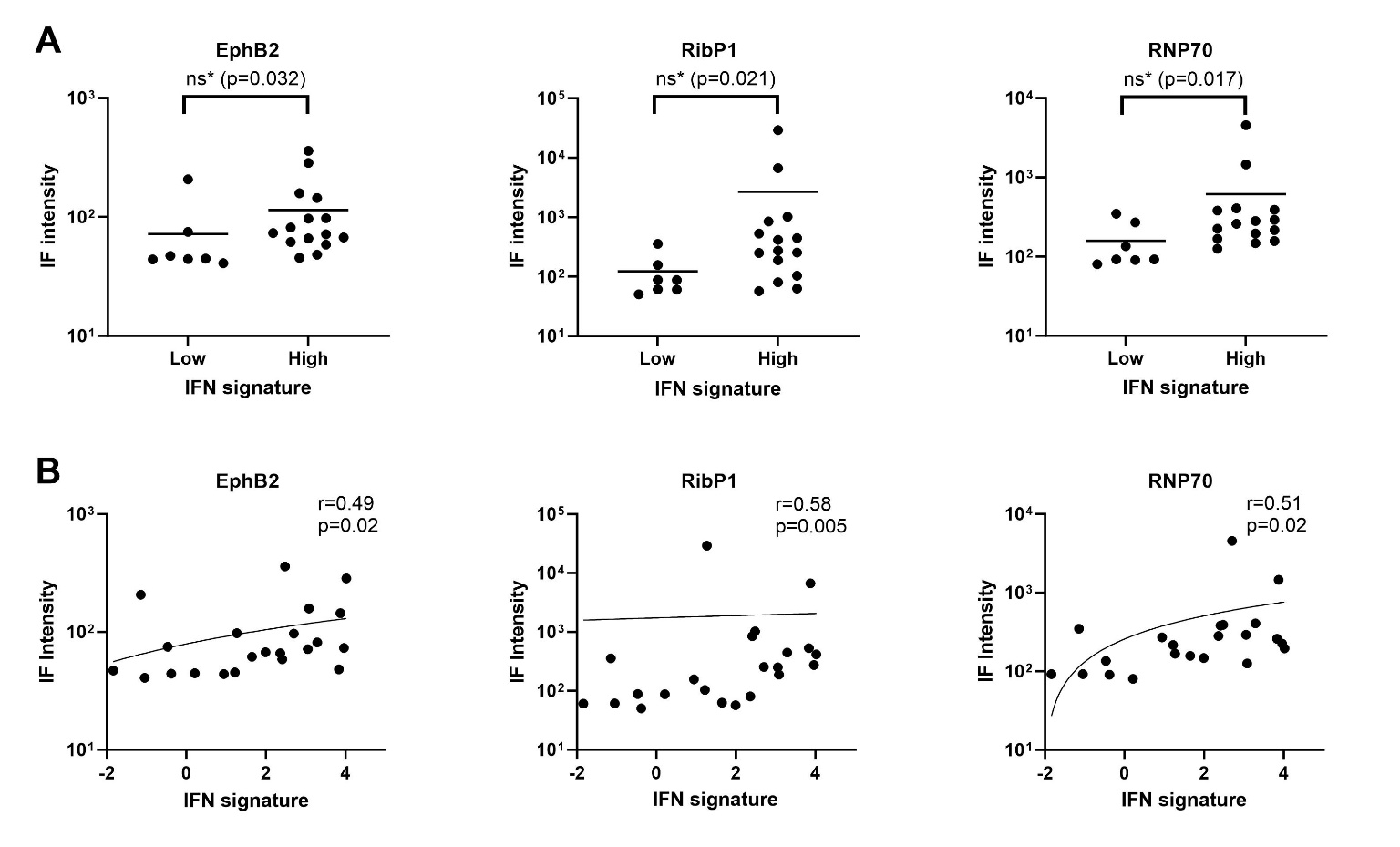


**Figure 3:** (**A)** Immunofluorescence (IF) intensity in IFN positive and IFN negative patiënts for EphB2, RipP1, and RNP70. Line represents mean IF intensity. **(B)** Correlation of EphB2, RipP1, and RNP70 IF intensity with IFN score.IF:immunofluorescence; IFN:interferon. *not significant after Bonferroni correction


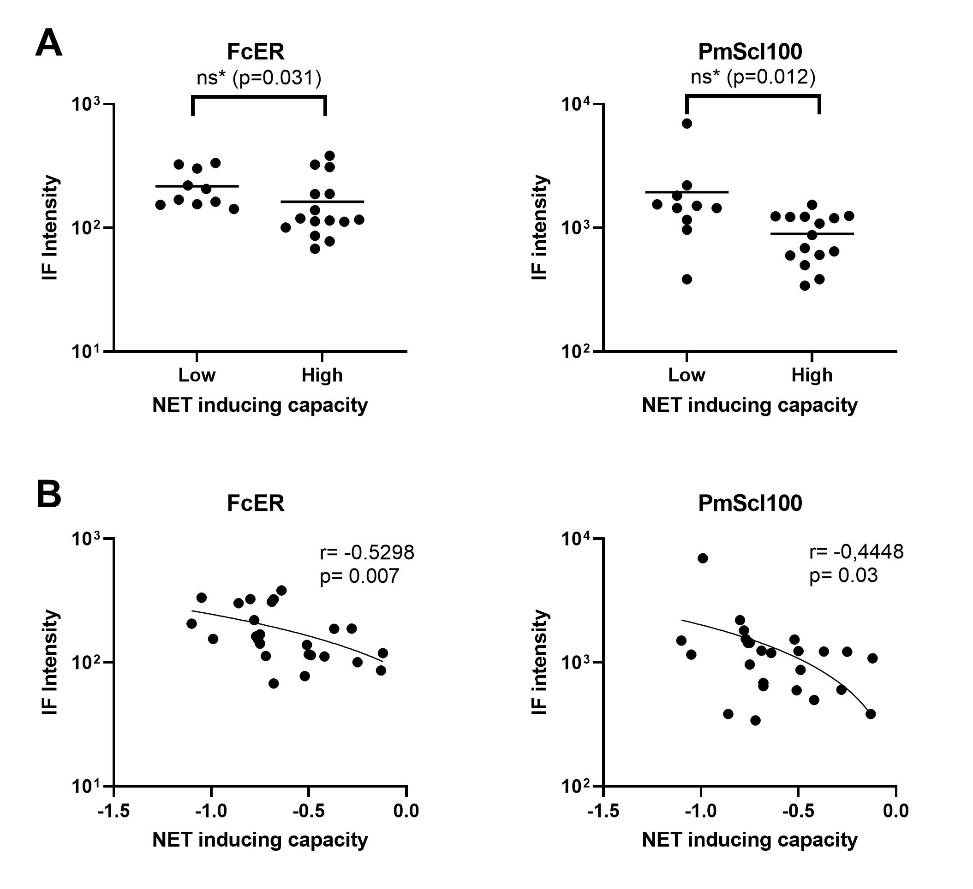


**Figure 4:** **(A)** Immunofluorescence (IF) intensity of autoantibodies against FcER and PmScl100 in patients with a low and high NET inducing capacity; Line represents mean IF intensity. **(B)** Correlation of FcER and PmScl100 IF intensity with NET inducing capacity. IF: immunofluorescence; NET: NETosis. *Not significant after Bonferroni correction for multiple testing.


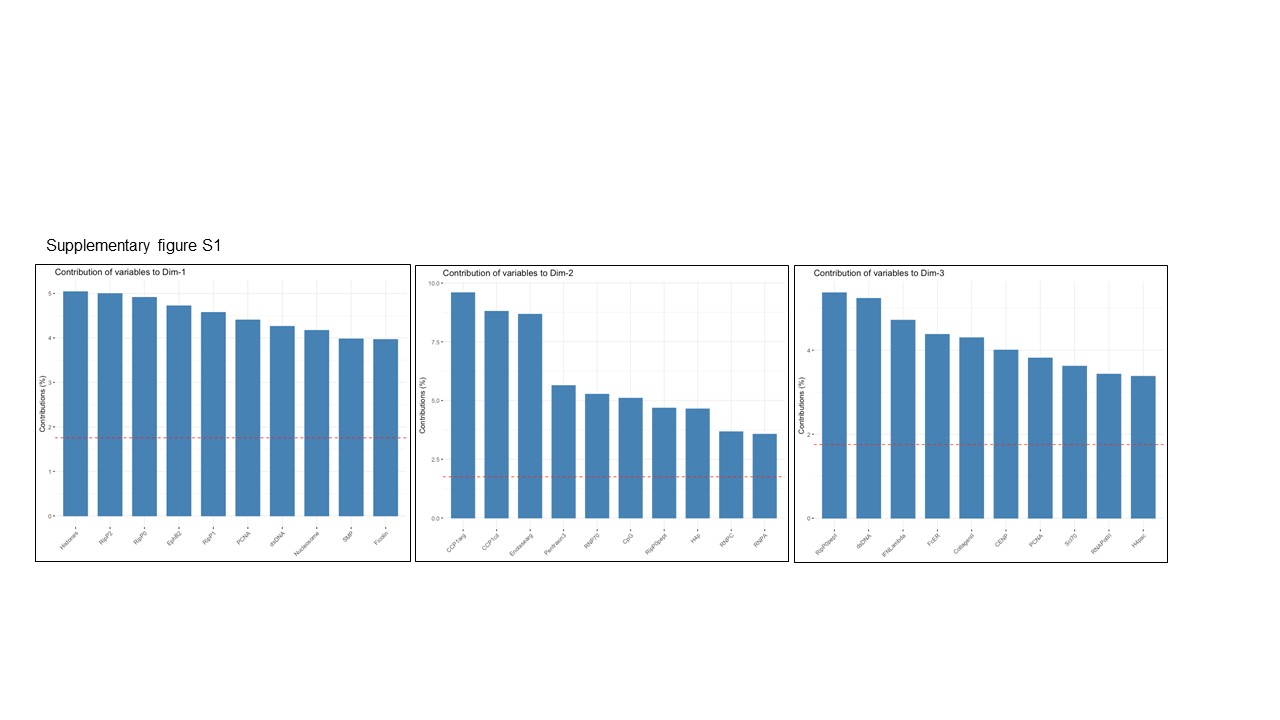
Supplementary figure S1: contribution of variables to PC1 (left), PC2 (middle) and PC3 (right).

**References**

[1] G. Stojan, M. Petri. Epidemiology of systemic lupus erythematosus: an update. Curr Opin Rheumatol, 2018;30**:**144-50.

[2] M. Gatto, M. Zen, L. Iaccarino, A. Doria. New therapeutic strategies in systemic lupus erythematosus management. Nat Rev Rheumatol, 2019;15**:**30-48.

[3] E. Rodriguez-Almaraz, E. Gutierrez-Solis, E. Rabadan, P. Rodriguez, L. Carmona, E. Morales, M. Galindo. Something new about prognostic factors for lupus nephritis? A systematic review. Lupus, 2021;30**:**2256-67.

[4] D. Toro-Dominguez, J. Martorell-Marugan, D. Goldman, M. Petri, P. Carmona-Saez, M. E. Alarcon-Riquelme. Stratification of Systemic Lupus Erythematosus Patients Into Three Groups of Disease Activity Progression According to Longitudinal Gene Expression. Arthritis Rheumatol, 2018;70**:**2025-35.

[5] S. N. Kariuki, B. S. Franek, A. A. Kumar, J. Arrington, R. A. Mikolaitis, T. O. Utset *et al.* Trait-stratified genome-wide association study identifies novel and diverse genetic associations with serologic and cytokine phenotypes in systemic lupus erythematosus. Arthritis Res Ther, 2010;12**:**R151.

[6] W. A. Haynes, D. J. Haddon, V. K. Diep, A. Khatri, E. Bongen, G. Yiu *et al.* Integrated, multicohort analysis reveals unified signature of systemic lupus erythematosus. JCI Insight, 2020;5.

[7] J. A. R. Guimaraes, S. D. C. Furtado, A. Lucas, B. Mori, J. F. M. Barcellos. Diagnostic test accuracy of novel biomarkers for lupus nephritis-An overview of systematic reviews. PLoS One, 2022;17**:**e0275016.

[8] M. J. Wahadat, D. Schonenberg-Meinema, C. G. van Helden-Meeuwsen, S. J. van Tilburg, N. Groot, E. J. H. Schatorje *et al.* Gene signature fingerprints stratify SLE patients in groups with similar biological disease profiles: a multicentre longitudinal study. Rheumatology (Oxford, England), 2022;61**:**4344-54.

[9] G. Barturen, S. Babaei, F. Catala-Moll, M. Martinez-Bueno, Z. Makowska, J. Martorell-Marugan *et al.* Integrative Analysis Reveals a Molecular Stratification of Systemic Autoimmune Diseases. Arthritis Rheumatol, 2021;73**:**1073-85.

[10] G. C. Tsokos, M. S. Lo, P. Costa Reis, K. E. Sullivan. New insights into the immunopathogenesis of systemic lupus erythematosus. Nat Rev Rheumatol, 2016;12**:**716-30.

[11] N. Marto, M. L. Bertolaccini, E. Calabuig, G. R. Hughes, M. A. Khamashta. Anti-C1q antibodies in nephritis: correlation between titres and renal disease activity and positive predictive value in systemic lupus erythematosus. Annals of the rheumatic diseases, 2005;64**:**444-8.

[12] E. J. ter Borg, G. Horst, E. J. Hummel, P. C. Limburg, C. G. Kallenberg. Measurement of increases in anti-double-stranded DNA antibody levels as a predictor of disease exacerbation in systemic lupus erythematosus. A long-term, prospective study. Arthritis Rheum, 1990;33**:**634-43.

[13] L. Bennett, A. K. Palucka, E. Arce, V. Cantrell, J. Borvak, J. Banchereau, V. Pascual. Interferon and granulopoiesis signatures in systemic lupus erythematosus blood. J Exp Med, 2003;197**:**711-23.

[14] E. C. Baechler, F. M. Batliwalla, G. Karypis, P. M. Gaffney, W. A. Ortmann, K. J. Espe *et al.* Interferon-inducible gene expression signature in peripheral blood cells of patients with severe lupus. Proc Natl Acad Sci U S A, 2003;100**:**2610-5.

[15] R. A. M. Furie, E.; Bruce, I.N.; Manzi, S.; Kalunian, K.C.; Vital, E.M. Type I interferon inhibitor anifrolumab in active systemic lupus erythematosus (TULIP-1): a randomised, controlled, phase 3 trial. The Lancet, 2019;1**:**E208-E19.

[16] E. F. Morand, R. Furie, Y. Tanaka, I. N. Bruce, A. D. Askanase, C. Richez *et al.* Trial of Anifrolumab in Active Systemic Lupus Erythematosus. The New England journal of medicine, 2020;382**:**211-21.

[17] G. S. Garcia-Romo, S. Caielli, B. Vega, J. Connolly, F. Allantaz, Z. Xu *et al.* Netting neutrophils are major inducers of type I IFN production in pediatric systemic lupus erythematosus. Sci Transl Med, 2011;3**:**73ra20.

[18] M. van der Linden, L. L. van den Hoogen, G. H. A. Westerlaken, R. D. E. Fritsch-Stork, J. A. G. van Roon, T. Radstake, L. Meyaard. Neutrophil extracellular trap release is associated with antinuclear antibodies in systemic lupus erythematosus and anti-phospholipid syndrome. Rheumatology (Oxford, England), 2018;57**:**1228-34.

[19] K. Akita, K. Yasaka, T. Shirai, T. Ishii, H. Harigae, H. Fujii. Interferon alpha Enhances B Cell Activation Associated With FOXM1 Induction: Potential Novel Therapeutic Strategy for Targeting the Plasmablasts of Systemic Lupus Erythematosus. Front Immunol, 2020;11**:**498703.

[20] K. Kiefer, M. A. Oropallo, M. P. Cancro, A. Marshak-Rothstein. Role of type I interferons in the activation of autoreactive B cells. Immunol Cell Biol, 2012;90**:**498-504.

[21] L. L. van den Hoogen, R. D. Fritsch-Stork, M. A. Versnel, R. H. Derksen, J. A. van Roon, T. R. Radstake. Monocyte type I interferon signature in antiphospholipid syndrome is related to proinflammatory monocyte subsets, hydroxychloroquine and statin use. Annals of the rheumatic diseases, 2016;75**:**e81.

[22] T. Brunekreef, M. Limper, R. Melchers, L. Mathsson-Alm, J. Dias, I. Hoefer *et al.* Microarray testing in patients with systemic lupus erythematosus identifies a high prevalence of CpG DNA-binding antibodies. Lupus Sci Med, 2021;8.

[23] B. Artim-Esen, E. Cene, Y. Sahinkaya, S. Ertan, O. Pehlivan, S. Kamali *et al.* Cluster analysis of autoantibodies in 852 patients with systemic lupus erythematosus from a single center. J Rheumatol, 2014;41**:**1304-10.

[24] P. Budde, H. D. Zucht, S. Vordenbaumen, H. Goehler, R. Fischer-Betz, M. Gamer *et al.* Multiparametric detection of autoantibodies in systemic lupus erythematosus. Lupus, 2016;25**:**812-22.

[25] K. H. Ching, P. D. Burbelo, C. Tipton, C. Wei, M. Petri, I. Sanz, M. J. Iadarola. Two major autoantibody clusters in systemic lupus erythematosus. PLoS One, 2012;7**:**e32001.

[26] L. M. Diaz-Gallo, V. Oke, E. Lundstrom, K. Elvin, Y. Ling Wu, S. Eketjall *et al.* Four Systemic Lupus Erythematosus Subgroups, Defined by Autoantibodies Status, Differ Regarding HLA-DRB1 Genotype Associations and Immunological and Clinical Manifestations. ACR Open Rheumatol, 2022;4**:**27-39.

[27] Y. Pacheco, J. Barahona-Correa, D. M. Monsalve, Y. Acosta-Ampudia, M. Rojas, Y. Rodriguez *et al.* Cytokine and autoantibody clusters interaction in systemic lupus erythematosus. J Transl Med, 2017;15**:**239.

[28] M. Y. Choi, I. Chen, A. E. Clarke, M. J. Fritzler, K. A. Buhler, M. Urowitz *et al.* Machine learning identifies clusters of longitudinal autoantibody profiles predictive of systemic lupus erythematosus disease outcomes. Annals of the rheumatic diseases, 2023;82**:**927-36.

[29] J. R. Doedens, W. D. Jones, K. Hill, M. J. Mason, V. H. Gersuk, P. J. Mease *et al.* Blood-Borne RNA Correlates with Disease Activity and IFN-Stimulated Gene Expression in Systemic Lupus Erythematosus. J Immunol, 2016;197**:**2854-63.

[30] J. Hua, K. Kirou, C. Lee, M. K. Crow. Functional assay of type I interferon in systemic lupus erythematosus plasma and association with anti-RNA binding protein autoantibodies. Arthritis Rheum, 2006;54**:**1906-16.

[31] Q. Z. Li, J. Zhou, Y. Lian, B. Zhang, V. K. Branch, F. Carr-Johnson *et al.* Interferon signature gene expression is correlated with autoantibody profiles in patients with incomplete lupus syndromes. Clin Exp Immunol, 2010;159**:**281-91.

[32] T. Lovgren, M. L. Eloranta, U. Bave, G. V. Alm, L. Ronnblom. Induction of interferon-alpha production in plasmacytoid dendritic cells by immune complexes containing nucleic acid released by necrotic or late apoptotic cells and lupus IgG. Arthritis Rheum, 2004;50**:**1861-72.

[33] F. Chasset, C. Ribi, M. Trendelenburg, U. Huynh-Do, P. Roux-Lombard, D. S. Courvoisier *et al.* Identification of highly active systemic lupus erythematosus by combined type I interferon and neutrophil gene scores vs classical serologic markers. Rheumatology (Oxford, England), 2020;59**:**3468-78.

[34] C. E. Weckerle, B. S. Franek, J. A. Kelly, M. Kumabe, R. A. Mikolaitis, S. L. Green *et al.* Network analysis of associations between serum interferon-alpha activity, autoantibodies, and clinical features in systemic lupus erythematosus. Arthritis Rheum, 2011;63**:**1044-53.

[35] S. Assassi, M. D. Mayes, F. C. Arnett, P. Gourh, S. K. Agarwal, T. A. McNearney *et al.* Systemic sclerosis and lupus: points in an interferon-mediated continuum. Arthritis Rheum, 2010;62**:**589-98.

[36] J. A. Reynolds, T. A. Briggs, G. I. Rice, S. Darmalinggam, V. Bondet, E. Bruce *et al.* Type I interferon in patients with systemic autoimmune rheumatic disease is associated with haematological abnormalities and specific autoantibody profiles. Arthritis Res Ther, 2019;21**:**147.

[37] L. Mozo, P. Lopez, L. Caminal-Montero, J. Rodriguez-Carrio, A. Suarez. Anti-ribosomal P antibodies are associated with elevated circulating IFNalpha and IL-10 levels in systemic lupus erythematosus patients. Lupus, 2014;23**:**1477-85.

[38] M. A. Smith, C. C. Chiang, K. Zerrouki, S. Rahman, W. I. White, K. Streicher *et al.* Using the circulating proteome to assess type I interferon activity in systemic lupus erythematosus. Sci Rep, 2020;10**:**4462.

[39] A. Sakamoto, H. Ishibashi-Ueda, Y. Sugamoto, T. Higashikata, S. Miyamoto, M. A. Kawashiri *et al.* Expression and function of ephrin-B1 and its cognate receptor EphB2 in human atherosclerosis: from an aspect of chemotaxis. Clin Sci (Lond), 2008;114**:**643-50.

[40] D. Vreeken, C. S. Bruikman, S. M. L. Cox, H. Zhang, R. Lalai, A. Koudijs *et al.* EPH receptor B2 stimulates human monocyte adhesion and migration independently of its EphrinB ligands. J Leukoc Biol, 2020;108**:**999-1011.

[41] D. F. Azzouz, G. V. Martin, F. Arnoux, N. Balandraud, T. Martin, S. Dubucquoi *et al.* Anti-Ephrin Type-B Receptor 2 (EphB2) and Anti-Three Prime Histone mRNA EXonuclease 1 (THEX1) Autoantibodies in Scleroderma and Lupus. PLoS One, 2016;11**:**e0160283.

[42] K. Tselios, D. D. Gladman, J. Su, O. Ace, M. B. Urowitz. Evolution of Risk Factors for Atherosclerotic Cardiovascular Events in Systemic Lupus Erythematosus: A Longterm Prospective Study. J Rheumatol, 2017;44**:**1841-9.

[43] M. Bruschi, A. Bonanni, A. Petretto, A. Vaglio, F. Pratesi, L. Santucci *et al.* Neutrophil Extracellular Traps Profiles in Patients with Incident Systemic Lupus Erythematosus and Lupus Nephritis. J Rheumatol, 2020;47**:**377-86.

[44] E. Fiebiger, F. Hammerschmid, G. Stingl, D. Maurer. Anti-FcepsilonRIalpha autoantibodies in autoimmune-mediated disorders. Identification of a structure-function relationship. J Clin Invest, 1998;101**:**243-51.

[45] K. Hanke, C. S. Bruckner, C. Dahnrich, D. Huscher, L. Komorowski, W. Meyer *et al.* Antibodies against PM/Scl-75 and PM/Scl-100 are independent markers for different subsets of systemic sclerosis patients. Arthritis Res Ther, 2009;11**:**R22.
